# An overview of the risk factors for producing fifth metatarsal fracture in sports activities: A systematic review

**DOI:** 10.1002/jfa2.70012

**Published:** 2024-10-18

**Authors:** Luis Angel Ortiz‐Lango, Israel Miguel‐Andrés, Daniel López‐López, José de Jesús Mayagoitiza‐Vázquez, Ricardo Becerro‐de‐Bengoa‐Vallejo, Marta Losa‐Iglesias, Juan Gómez‐Salgado, Miguel Ángel Saavedra‐García

**Affiliations:** ^1^ Laboratorio Nacional CONAHCYT en Biomecánica del Cuerpo Humano CIATEC, A.C. León Guanajuato Mexico; ^2^ Research, Health and Podiatry Group Department of Health Sciences Faculty of Nursing and Podiatry Industrial Campus of Ferrol Universidade da Coruña Ferrol Spain; ^3^ Biomecánica, Centro de Innovación Aplicada en Tecnologías Competitivas León Guanajuato México; ^4^ Faculty of Nursing, Physiotherapy and Podiatry Universidad Complutense de Madrid Madrid Spain; ^5^ Faculty of Health Sciences Universidad Rey Juan Carlos Alcorcón, Madrid Spain; ^6^ Department of Sociology, Social Work and Public Health Universidad de Huelva Huelva Spain; ^7^ Safety and Health Postgraduate Programme Universidad Espíritu Santo Guayaquil Ecuador; ^8^ Group of Research in Sport Science (INCIDE) Department of Physical Education and Sport Universidade da Coruña A Coruña Spain

**Keywords:** fifth metatarsal fracture, foot, risk factors, sport

## Abstract

**Introduction:**

The fifth metatarsal fracture is a foot injury that occurs in sports activities. This fracture has been associated with risk factors based on intrinsic variables such as type of feet (flatfoot or cavus foot), foot pathologies, and bone density among others. Extrinsic variables associated with fifth metatarsal fractures include sports maneuvers, the type of sports practice, and contact surface. Although this injury has been investigated over the years, there is no consensus on the most relevant risk factors that cause this injury. An increase in the number of people with fractures makes it a relevant topic of research. The objective of this review was to identify an overview of the risk factors for producing the fifth metatarsal fracture based on intrinsic and extrinsic variables in sports activities. Furthermore, this review aimed to clarify what is known and what is needed on the risk factors that can influence the appearance of the fracture.

**Methods:**

A search in electronic databases, such as Scopus (*n* = 87), PubMed (*n* = 187), and Web of Science (*n* = 173) was conducted. The initial search yielded 447 titles and abstracts, from which 31 papers were selected for detailed analysis after screening all citations against the eligibility criteria.

**Results:**

After screening the manuscripts, it was found that the fifth metatarsal fracture can be produced by multiple factors. However, most of the studies focus on one or two specific risk factors. It was found that soccer (38.7%) is the sports activity that presents a higher risk of getting a fifth metatarsal fracture compared to other sports activities. The second risk factor was the performance of critical maneuvers (22.5%) and the third one was the biomechanics of the foot (22.5%).

**Conclusion:**

It is paramount to identify the most critical risk factors linked to the fifth metatarsal fracture to be able to implement effective treatments and prevention strategies.

## 1 INTRODUCTION

The fifth metatarsal fracture is a common foot bone injury, which occurs when the fifth metatarsal bone breaks. It is an injury that can occur in people of all ages and levels of physical activity [1, 2]. As described by Sir Robert Jones in 1902, the fracture of the fifth metatarsal bone can be produced by a sprain of the foot. This injury has been investigated for many years, and it can be classified according to the location of the fracture. The fracture location zone mostly uses the Lawrence and Botte's classification [3, 4]. It describes three zones: zone 1, the tuberosity; zone 2, the metaphyseal–diaphyseal junction; and zone 3, the diaphyseal area within 1.5 cm of the tuberosity. Fractures through zone 1 are called pseudo‐Jones fractures, and fractures through zone 2 are called Jones fractures [3, 4, 5, 6].

Symptoms of a fifth metatarsal fracture may include pain in the lateral part of the foot, swelling, and tenderness to touch. In some cases, the person may also have trouble walking or bearing on the affected foot [4, 5, 6, 7]. In addition, the treatment for a fifth metatarsal fracture depends on the severity of the injury. In mild cases, it may be enough to rest the foot and apply ice and compression to reduce swelling and pain. In more severe cases, it may be necessary to use a cast or splint to hold the bone in place while it heals. In some cases, surgery may be necessary to correct the fracture [1, 2, 4, 6, 8].

In sports, this injury is quite common, and it can be caused by a variety of factors based on intrinsic and extrinsic variables, especially in athletes who play high‐impact sports, and involve sudden changes in direction and repetitive movements of the foot, such as soccer, basketball, running, martial arts, and rugby [2, 9]. The intrinsic factors are those related to the anthropometry and physical condition of the people, such as the type of foot, density of the bone tissue, malformations, angular deviations of the bones, and so on. Conversely, the extrinsic factors are not related to the anthropometry or physical condition of the people, these risk factors are produced by high impact loads in the foot, change of direction (cutting motions), falls, footwear (soccer boots increase plantar loadings compared with running shoes [10]), insoles (different designs and materials such as carbon fiber [11]), or wrong physical execution (do not adhere correctly to the technical gesture of the sports activity [12]). In the athlete population, there has been an increment of participants with a fifth metatarsal fracture [1].

The incidence of the fifth metatarsal fracture varies according to the sport and the age of the athletes [2, 3]. In recent studies, fracture of the fifth metatarsal accounts for 10% of all sports injuries and 25% of foot injuries, with a higher incidence in men than in women, and occurring more frequently in the dominant leg [2, 4]. Another risk factor associated with the fracture of the fifth metatarsal is the sudden change of direction [6].

Another study shows that the morphology of the foot, especially the shape (varus/valgus foot alignment) can influence the appearance of the fracture of the fifth metatarsal [13]. For example, athletes with flat feet are at a higher risk of suffering this injury due to an increased load on the outside of the foot. The type of feet is a structural condition where the plantar vault is modified. The modification of this structure could affect the plantar pressure distribution and become a risk factor for the fifth metatarsal fracture [14, 15]. The type of feet can be detected through the analysis of the morphology of the soles of feet as described by Ramos‐Frutos et al. [16]. A higher incidence has also been found in athletes with inadequate running technique, such as an excessive pronation pattern [8]. Plantar pressure asymmetries have been considered a risk factor for producing fifth metatarsal fracture. Azevedo et al., found that young soccer players present higher pressure values in the fifth metatarsal bone and the hallux in the non‐preferred foot during static conditions. This could be a risk factor for producing stress fracture of the fifth metatarsal bone [9, 17].

Although there is relevant information on the incidence of fracture and pathology in sports, there is no consensus in the scientific community on the risk factors (intrinsic and extrinsic variables) in sports associated with the appearance of this injury. A systematic review was conducted to systematically map the intrinsic and extrinsic variables to produce the fifth metatarsal fractures, as well as to identify any existing gaps in knowledge of this injury. The following research question was formulated: What is known from the literature about the risk factors for producing fifth metatarsal fracture based on intrinsic and extrinsic variables in the athlete population?

## 2 MATERIALS AND METHODS

### 2.1 Study design

The protocol was written using as reference the guideline of the Cochrane Collaboration [18] and the PRISMA 2020 statement: an updated guideline for reporting systematic reviews [19], file number CRD42024520696.

### 2.2 Inclusion and exclusion criteria

The following inclusion and exclusion criteria were considered to determine which investigations should be included in the review. As inclusion criteria, peer‐reviewed journal papers were included if they were related to the risk factors of the fifth metatarsal fracture, published between the period of 2000–2023, written in English or Spanish, involved observational studies with sawbones models or finite element method, randomized clinical trials and controlled clinical trials, biomechanics of foot and pathologies, and stress fractures of the foot in sports. Papers were excluded if they did not fit into the main conceptual framework of the study, letters to the editor, commentaries, expert opinions, editorials, and other non‐original studies, case studies, or case reports, papers presenting treatment, management, and surgical procedures, and research published in other languages different from Spanish or English.

### 2.3 Search strategy

To identify potentially relevant documents, the following electronic databases were searched from 2000 to 2023: Scopus (*n* = 173), PubMed (*n* = 87), and Web of Science (*n* = 187). The search strategies were created to achieve a high‐sensitivity search, the terms indicated were collected and sorted into the search equation shown in Table 1. The search equation gave a total of 447 papers from the different databases. The search was conducted in 2023.

**TABLE 1:** Search equation used for the literature review.

Search equation
*Fifth* AND *metatarsal*
AND *Fracture* *AND* *(Sport OR risk AND factors)*
AND NOT (“case study” OR “Case report” OR surgical)

We considered the following definitions: associated factors refer to variables correlated with a particular outcome or condition but do not necessarily have a causal relationship. On the other hand, risk factors are variables associated with an increased likelihood of developing the outcome or condition being studied. However, for this research, the revision evidenced that the scientific community does not distinguish between risk and associated factors for the incidence of fracture of the fifth metatarsal. Then, all factors were considered as risk factors even though some of them are associated factors by definition.

**FIGURE 1:**
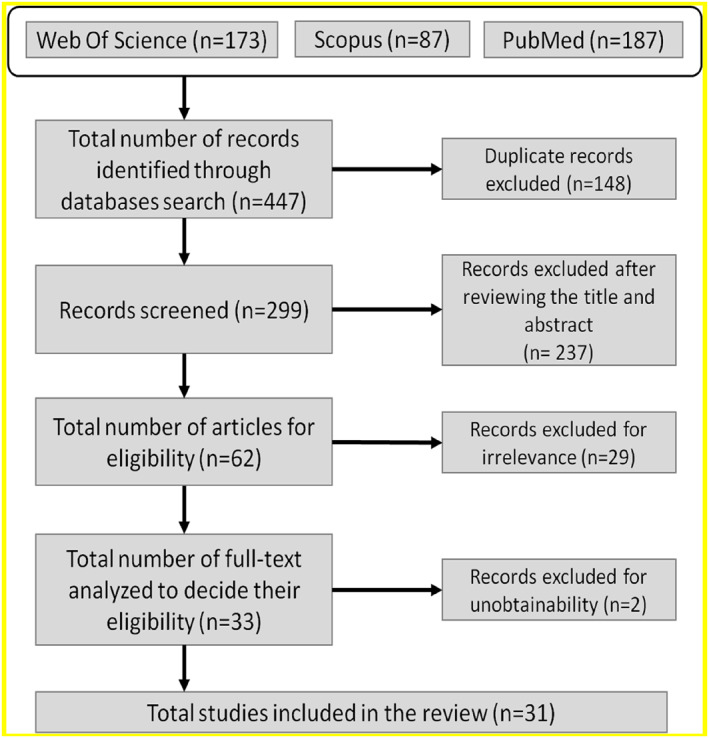
Search method flow diagram created based on the Guideline Cochrane Collaboration [18] and PRISMA methodology [19].

Two authors reviewed the search findings related to the risk factors of the fifth metatarsal fracture. If the outcomes were not clear during the data extraction, the whole research group participated to have consensus. All references were manually evaluated, and those manuscripts that might be incorporated were also reviewed and obtained. After reviewing the three databases, 148 duplicate manuscripts were removed, having a number of 299 articles for the analysis. The title and abstract of the remaining articles were reviewed, and then, 237 articles were excluded because they were not fully related to the aim of the review. Finally, the process of selecting papers remained in this systematic review with 33 articles. However, due to the unobtainability of two articles, a total of 31 records were considered for deeper analysis, Figure 1.

### 2.4 Synthesis of information and management of search results

Two reviewers screened all publications, discussed the results, and planned the screening for this review. Moreover, if there were doubts about something, any research regarding the inclusion and exclusion criteria was cleared up by all authors. All references added were manually evaluated, and the articles that were incorporated were also reviewed and obtained. A data‐charting form was developed by all reviewers to determine which variables to extract from the manuscripts. Then, two reviewers independently reviewed the articles, discussed the results, and continuously updated the data‐charting form by an iterative process. In addition, the risk of bias assessment was analyzed using the tool Review Manager (RevMan) of the Cochrane Library.

## 3 RESULTS

### 3.1 Summary of the investigations

A total of 4118 cases were studied, from experimental analysis with test subjects, retrospective case studies, and evaluations in cadaveric foot specimens. Concerning the type of studies found, most of them were observational, experimental, and retrospective or longitudinal cohort studies. The range of ages of the subjects considered in the studies was between 19 and 30 years.

From the analysis of the review, 12 articles (38.7%) agree that the practice of soccer is the main factor associated with the appearance of the fracture. This sports activity is the most practiced around the world, the foot supports too much stress, and this could represent a risk factor for producing the fifth metatarsal fracture [2, 9, 17, 20, 21, 22, 23, 24, 25, 26, 27, 28]. On the other hand, seven articles (22.5%) state that the fracture could be related to the performance of sports maneuvers [9, 27, 29, 30, 31, 32, 33], three (9.6%) with the footwear (soccer boots increase plantar loadings compared with running shoes) [9, 10, 20], four (12.9%) with the phases of gait (toe off phase) [11, 22, 34, 35], seven (22.5%) with the biomechanics of the foot (interactions of loadings with the structure or musculoskeletal disorders of the foot) [20, 22, 26, 36, 37, 38, 39], one (3.2%) with the anatomy of the fifth metatarsal bone [21], two (6.4%) associated with the range of motion of the hip (internal rotation) [20, 40], two (6.4%) with the playing surface (synthetic or natural field) [20, 41], one (3.2%) with the anthropometric characteristics of the subject [20], two (6.4%) with the use of orthoses or insoles (different designs and materials such as carbon fiber) [11, 33], one (3.2%) to the age [23], one (3.2%) to the anatomy of the soft tissue connected to the fifth metatarsal bone [42], four (12.9%) to foot morphology [26, 43, 44, 45], and one (3.2%) to ankle morphology [24].

Technical aspects of the articles investigated show a high incidence of research groups toward measurements with plantar pressure to determine the risk of presenting a fracture of the fifth metatarsal. However, other studies use radiographic measurements to estimate the occurrence of this injury. The main findings of this systematic review are presented in Table 2 and the net chart in Figure 4.

**TABLE 2:** Systematic review of the risk factors of the fifth metatarsal fracture.

Author	Cases	Type of study	Type of fifth metatarsal fracture	Risk factors associated	Type of risk factor	Outcomes measures	Outcome
Azevedo et al. [17]	Thirty young adolescents were divided into a soccer player group (*n* = 15) or a matched control group (*n* = 15)	Observational	Stress fracture	Practice of soccer	Extrinsic	Asymmetries in the magnitude of plantar pressure	Higher pressure was found in the hallux, 5th metatarsal, and medial rearfoot of the non‐preferred foot in the young soccer players. The asymmetries observed in young soccer players suggest that they have specific adaptations that may result from mechanical demands during soccer practice
Azevedo et al. [9]	Twenty‐six male adolescents	Comparative	Not indicated	Sports maneuvers, footwear, and practice of soccer	Extrinsic	Foot sensitivity, ankle range of motion, Q‐angle, and plantar pressure	During the performance of soccer actions, young players showed higher peak pressure in the lateral region of the foot including the fifth metatarsal region
Carl et al. [10]	17 elite male soccer professionals	Case series	Not indicated	Footwear	Extrinsic	Changes of peak plantar pressure for 9 defined foot portions between soccer boots and running shoes	In running, soccer boots generate excessive foot loadings predominantly under the lateral midfoot, as compared with running shoes
Donahue et al. [34]	Fifteen pairs of feet from fresh‐frozen cadavers. (8 male and 7 female)	Experimental	Stress fracture (fifth metatarsal diaphysis)	Gait phases	Intrinsic	Microcrack density (Cr.Dn) and surface density (Cr.S.Dn)	The stance phase of gait has no influence on the appearance of fifth metatarsal stress fracture
Fleischer et al. [36]	Fifty patients with acute Jones fracture and 200 control were included	Retrospective, matched, case‐control	Jones fracture	Foot biomechanics	Intrinsic	Metatarsus adductus angle (MAA), 1st and 2nd intermetatarsal angle (1/2 IMA), 4th and 5th intermetatarsal angle (4/5 IMA), Meschans's metatarsal break angle, talo‐calcaneal angle (TCA), calcaneo‐cuboid abduction angle, talo‐1st metatarsal angle, and hallux abductus angle	Hindfoot position is not an important risk factor as forefoot posture. The effect of hindfoot varus was significantly lessened when also considering the effects of the forefoot. Patients with metatarsus adductus alone were at 2.4 times greater risk of presenting with a Jones fracture. The risk of Jones fracture increases with adducted forefoot posture
Fujitaka et al. [21]	60 feet from 30 university soccer players with Jones fracture and a control group of 60 feet from 60 male university soccer players without Jones fracture	Cohort study	Jones fracture	Practice of soccer and anatomy of fifth metatarsal bone	Extrinsic/intrinsic	Absolute length of the fifth metatarsal	Proximally longer fifth metatarsal may cause greater stress at the base of the fifth metatarsal bone. In addition, high medial longitudinal arch may contribute to increased load on the lateral side of the foot
Fujitaka et al. [20]	273 male athletes from the same college soccer team	Longitudinal study	Stress fracture	Practice of soccer, foot biomechanics, range of motion, footwear, playing surface and physical characteristics	Intrinsic/extrinsic	Measurements of stature, body weight, body mass index, foot‐arch height ratio, toe‐grip strength, quadriceps angle, leg‐heel angle, functional reach test, single‐leg standing time with eyes closed, straight‐leg, raise angle, finger‐floor distance, heel‐buttock distance, ankle joint range of motion, and a general joint laxity test. Survey of living environment: History of sports injuries, dominant leg, footwear, playing surface	A weak toe‐grip is associated with a fifth metatarsal stress fracture. In addition, fifth metatarsal stress fractures were more common in the nondominant leg
Gu et al. [35]	3 cases	Finite element study	Not indicated	Gait phases	Intrinsic	Von Mises stress distribution and concentration within the metatarsals and plantar pressure distribution	The peak stress point was found to be near the proximal part of the fifth metatarsal with 20° inversion angle
Hetsroni et al. [22]	Ten injured professional soccer players and 10 control soccer players	Comparative	Unilateral proximal fifth metatarsal stress fracture	Gait phases, practice of soccer and foot biomechanics	Intrinsic/extrinsic	Static evaluation of foot and arch structure. And dynamic plantar foot pressure	Static measurements of foot and arch structure did not reveal differences among groups. However, plantar pressure revealed pressure reduction only in the injured limbs of injured athletes
Hoffman et al. [11]	10 midtibial cadaveric specimens	Controlled laboratory study	Stress fracture	Use of orthotic and gait phases	Extrinsic/intrinsic	Peak tensile strain in zone II and zone III of fifth metatarsal	The use of orthotic devices reduced principal strain relative to the condition of sneakers without any orthosis in zone II and zone III
Hotfiel et al. [23]	65 elite soccer players	Cross‐sectional study	Stress fracture	Age	Intrinsic	Peak plantar pressure in each region of the foot	A study across soccer players concludes asymmetrical plantar loading patterns with peak pressures under the lateral midfoot
Hunt and Goeb [29]	Sixteen elite, competitive athletes (8 with a history of Jones fracture, 8 without a history of foot injury)	Comparative	Jones fracture	Sport maneuvers	Extrinsic	Peak pressure, mean pressure, maximum force, and force‐time integral	Athletes with a history of Jones fracture exert significantly increased peak and mean forces at the base of the fifth metatarsal during common athletic activities
Kaneko et al. [42]	102 legs from 55 Japanese cadavers	Descriptive laboratory study	Jones fracture	Anatomy of tissue	Intrinsic	Type classification: Type I, attached to proximal to the border between zones 1 and 2; type IIa, attached to the border between zones 1 and 2 with one attached part; and type IIb, attached across the border between zones 1 and 2 with two or more attached parts. In comparison with footprint areas of the PB, PT, LB and LPL	Type I, which attaches proximal to zone 2, occurs with PB and LB. This finding suggested that type I is involved in traction stress
Karnovsky et al. [43]	Ninety‐six feet (51 athletes)	Case‐control study	Fractures of the proximal fifth metatarsal	Foot morphology	Intrinsic	Fourth‐fifth intermetatarsal angle and metatarsal angle were measured in an anteroposterior radiograph; the Meary's angle, talocalcaneal angle, and calcaneal pitch were measured in the lateral radiograph	Individuals with long, narrow and straight fifth metatarsal with an adducted forefoot were most at risk for fifth metatarsal fractures
Kizaki et al. [24]	15 cases of stress fractures of the proximal diaphysis of the fifth metatarsal, 85 controls	Retrospective case‐control	Proximal diaphyseal stress fractures	Ankle morphology and practice of soccer	Intrinsic/extrinsic	The measure of medial malleolar slip angle (MMSA), the ratio of the medial malleolar length to the width of the talar dome (MML:TD ratio), the ratio of the lateral malleolar length to the width of the TD (LML:TD ratio) and the ratio of the MML to the LML (MML:LML ratio)	The study shows that a wide MMSA (measure medial malleolar slip angle) was associated with a proximal diaphyseal stress fracture of the fifth metatarsal in professional soccer players
Kuzuyama et al. [25]	Fifty‐one male soccer players (31 professional, 20 high‐school)	Retrospective comparative study	Proximal fifth metatarsal fracture	Practice of soccer	Extrinsic	Measure of plantar pressure patterns	The results of this study revealed that players with excessive loading in the lateral areas of the foot while walking have a risk of developing proximal fifth metatarsal fractures
Lee et al. [44]	50 consecutive athletes with a diagnosis of fifth metatarsal stress fracture and a control group matched for sport type and age	Comparative	Fifth metatarsal stress fracture	Foot morphology	Intrinsic	Fifth metatarsopha‐langeal (MTP‐5) angle, fourth‐fifth intermetatarsal (IMA4–5) angle, fifth metatarsal lateral deviation (MT5‐LD) angles were measured on standing antero‐posterior (AP) radiographs. Talo first metatarsal (T‐MT1) angle, talo‐calcaneal (TC) angle, and calcaneal pitch (CP) angle was measured on a standing lateral view, and MT5‐LD angle was measured on a 30‐degree medial oblique view	Fifth metatarsal stress fractures were found to be associated with elevated T‐MT1 angle and CP angle representing a cavus foot and the increased curvature of fifth metatarsal. In addition, the extent of fifth metatarsal curvature on a 30‐degree medial oblique view was found to be more related to the risk of fracture than the AP view
Matsuda et al. [26]	335 collegiate male soccer players	Case‐control study	Fifth metatarsal stress fracture	Foot biomechanics, foot morphology, and practice of soccer	Intrinsic/extrinsic	The foot length, arch height, weight‐bearing leg–heel alignment non‐weight‐bearing leg–heel alignment, forefoot angle relative to the rearfoot, forefoot angle relative to the horizontal axis, and foot pressure	Playing a midfield position and everted rearfoot and inverted forefoot alignments were associated with 5‐MT fractures
Miyamori et al. [41]	1854 football players, of which 41 experienced MT‐5 within the past 24 months	Retrospective cohort study	Fifth metatarsal stress fracture	Playing surface	Extrinsic	Baseline demographic data and the percentage of time spent playing on artificial turf and clay fields	A higher percentage of playing time on synthetic turf was a risk factor for developing MT‐5 in football players
Miyazaki et al. [27]	Five collegiate soccer players	Finite element study	Fifth metatarsal stress fracture	Sports maneuvers and practice of soccer	Extrinsic	Three‐dimensional foot kinematics, ground reaction force, and plantar pressure distribution. In addition, the strain distribution in the foot	The results showed that the mechanism of strain generation at the frequent fracture site varied depending on the type of cutting motion
Orendurff et al. [30]	10 college‐aged male athletes	Biomechanical analysis of stresses	Not indicated	Sport maneuvers	Extrinsic	The pressure differential between the base and head of the fifth metatarsal using a Pedar insole system	It appears that acceleration maneuvers (20 ± 13.1 N/cm^2^; *p* < 0.0001) may apply the largest bending moments to the fifth metatarsal and could lead to stress fractures
Raikin et al. [37]	Twenty‐one primary Jones fractures (20 patients)	Case series	Jones fracture	Foot biomechanics	Intrinsic	Radiographic measurements. Mean calcaneal pitch angle and Meary's angle	The pitch angle was 28.5° and the Meary angle was 13° convex upward in 18 of 21 hindfeet to be in varus
Riegger et al. [38]	Eight studies in the qualitative analysis (296 patients), and 5 in the quantitative synthesis (132 patients)	Systematic review and meta‐analysis	Jones fracture	Foot biomechanics	Intrinsic	Literature findings using mean difference of the assessed angles as an outcome measure (metatarsus adductus angle [MAA]), calcaneal pitch angle (CP), and Talo‐1rst metatarsus angle/Meary's angle (T1stMA)	Metatarsus adductus deformity seems to be correlated with a higher risk of proximal metatarsal fractures and Jones fractures
Saita et al. [40]	Japanese professional football players. 20 with and 40 without a history of Jones fracture	Case–controlled study	Jones fracture	Range of motion	Intrinsic	Univariate and multivariate logistic regression controlling for age, leg dominance, and body mass index	The restriction of HIR was associated with an increased risk of developing a Jones fracture
Shuen et al. [2]	791 patients with metatarsal fractures	Prospective cohort study	Metatarsal fractures	Practice of soccer	Extrinsic	Patients with metatarsal fractures were identified	Approximately 9.4% of metatarsal fractures were sustained through sport with soccer being the most associated
Sims et al. [28]	Thirty‐four athletes (17 men, 17 women)	Controlled laboratory study	Fifth metatarsal stress fractures	Gender and practice of soccer	Intrinsic	Contact area, maximum force, and the force‐time integral in the medial and lateral midfoot, medial, middle, and lateral forefoot as well as the hallux. A univariate ACOVA was performed on each dependent variable	The results of this study indicate that the increase in plantar loading on the lateral portion of the midfoot and forefoot in men could be one possible explanation for the increased incidence of fifth metatarsal stress fracture
Thomson et al. [31]	Fourteen elite male soccer players	Comparative study	Stress fracture	Sport maneuvers	Extrinsic	Plantar loading	After MT‐5 stress fracture, football players display significantly higher maximum plantar force at the lateral forefoot and lateral toes
Wilzman et al. [32]	A total of 39 elite collegiate runners (24 male, 15 female)	Cohort study	Bone stress injury	Sport maneuvers	Extrinsic	Plantar pressures and contact areas in 7 key areas of the foot	The models collectively suggested that higher plantar pressure may contribute to the risk of bone stress injury
Yoho et al. [45]	Review of 14 Jones fractures	Retrospective study	Jones fractures	Foot biomechanics	Intrinsic	Degree of metatarsus adductus (MMA)	A hindfoot varus attitude increases the risk of a Jones fracture
Yoho et al. [45]	Anteroposterior radiographs of 30 acute Jones fractures were compared with radiographs of 30 asymptomatic control subjects	Retrospective study	Jones fracture	Foot morphology	Intrinsic	Radiographic metatarsus angle measurements	Recognizing transverse plane forefoot pathology as a risk factor for the Jones fracture
Yu et al. [33]	14 male subjects	Preintervention and post‐intervention, repeated‐measures experimental design	Proximal fracture of the fifth metatarsal	Use of orthotic	Extrinsic	Ankle inversion angle and plantar forces and pressures on the fifth metatarsal during landing	Generic use of off‐the‐shelf foot orthoses with medial arch support causes increased plantar forces and pressures on the fifth metatarsal and may increase the risk for proximal fracture of the fifth metatarsal

Abbreviations: HIR, hip internal rotation; LB, lateral band of the plantar aponeurosis; LPL, long plantar ligament; PB, peroneus brevis muscle; PT, peroneus tertius.

**FIGURE 2:**
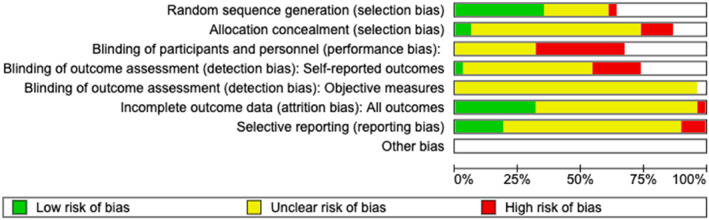
Analysis of the risk of bias of the studies included. (1) Green (low risk), (2) yellow (unclear risk), and (3) red (high risk).

**FIGURE 3:**
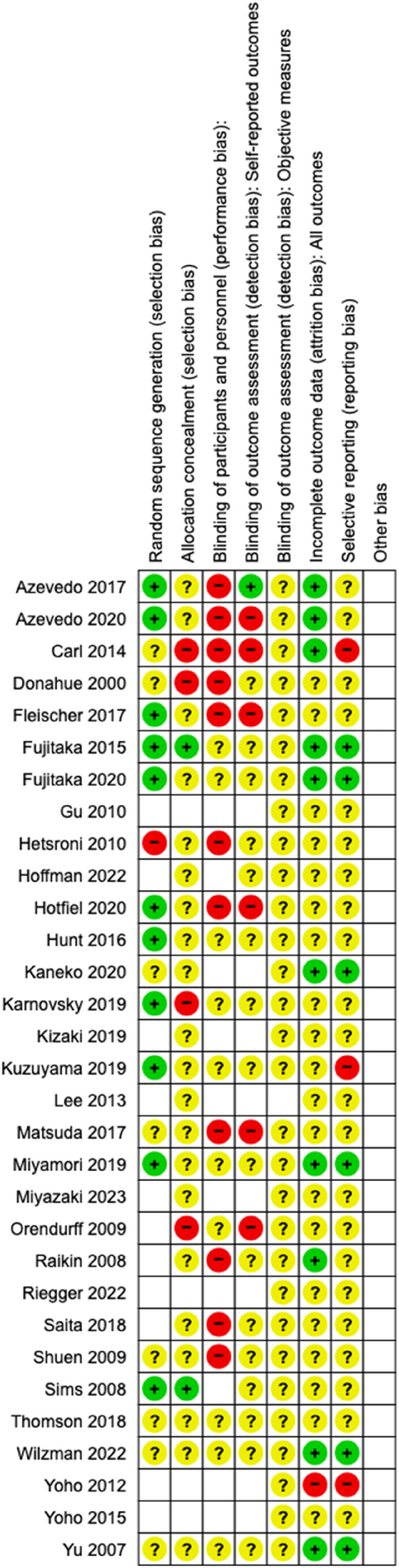
Risk of bias summary in the studies. (1) Green (low risk), (2) yellow (unclear risk), and (3) red (high risk).

The level of risk of bias of the 31 articles was performed in the Review Manager (RevMan) system of the Cochrane Library (version v.5.5). Few studies presented a low risk of bias (green color), in most of the cases, evaluating the risk of bias was not possible or the information was not clear (yellow color). Few of the articles presented a clear high risk of bias (red color). All data are shown in Figures 2 and 3. The meta‐analysis was not possible to perform because of the heterogeneity of the incorporated works.

**FIGURE 4:**
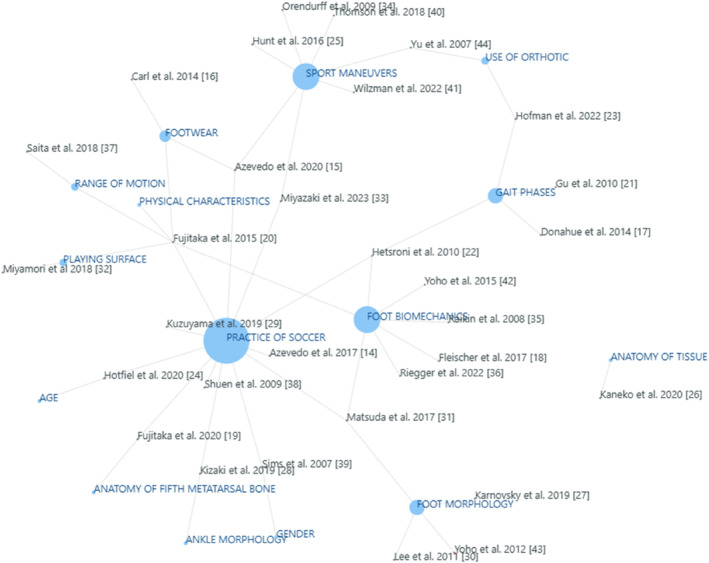
Network chart of the intrinsic and extrinsic risk factors of the fifth metatarsal fracture.

A more in‐depth analysis of the results allows us to observe that although the implications of various factors have indeed been observed, there is still a lack of knowledge in the understanding and classification of the risk factors of the fifth metatarsal fracture. Figure 4 shows the findings in the research related to intrinsic and extrinsic risk factors associated with the appearance of the fifth metatarsal fracture. It can be observed that no information reports the kinematic effect of sports maneuvers in the stress of the fifth metatarsal bone. It is known that sports maneuvers affect the plantar pressure of the foot, but nobody reports the kinematics parameters and the biomechanics of the foot that could be risk factors for the incidence of the fifth metatarsal fracture [2, 9, 17]. It was found that soccer, sport maneuvers, and foot biomechanics were the most relevant risk factors for the fifth metatarsal fracture, Figure 4.

## 4 DISCUSSION

In the network diagram shown in Figure 4, it can be seen that in reality, the fracture is due to the combination of multiple factors as previously mentioned. This implies that the prevalence of this injury is multifactorial. The studies analyzed in this systematic review show a combination of several risk factors, the most studied being the practice of soccer, sports maneuvers, and foot biomechanics.

To the best of our knowledge, this work is a novel systematic review in the analysis of the risk factors of the fifth metatarsal fracture. Although there are several studies about the fracture, most of them focus on a single or particular risk factor [9, 10, 17, 23, 25, 27, 28, 29, 30, 31, 32, 33, 34, 36, 37, 38, 39, 40, 41, 42, 43, 44, 45]. This study focused on the relevant published literature that exclusively evaluates the available scientific knowledge on the risk factors associated with the occurrence of fifth metatarsal fracture. We have found that fractures of the base of the fifth metatarsal are common in athletes in general and that they constitute a significant proportion of foot and ankle fractures. Furthermore, these fractures are associated with a prolonged recovery and a high rate of refractures, which affects time to get back to sport and work. Most studies have focused on the treatment of these fractures in athletes, but few have investigated the prevalence in the general population [1, 2, 6, 31].

Due to the apparent heterogeneity evaluated between risk factors and fracture incidence, the type of fracture, the population studied, the outcome measures, and the analysis of available studies with different risk factors, the investigations can be classified based on whether these factors depend on the morphological and anatomical conditions of the subject (intrinsic variables) or if they depend on external conditions (extrinsic variables). It was detected that the research showed a greater relationship with the intrinsic variables, without leaving aside the extrinsic variables. In this sense, it is important to note that there is no consensus among researchers to define the most critical risk factor for producing the fifth metatarsal fracture. Therefore, it is evident that this injury occurs due to multiple factors, and in some cases, the intrinsic factors could be more significant.

Based on the findings of Shuen et al., it was found that among the athletes who were presented the fifth metatarsal fracture, the players of soccer were the ones who had higher rates of incidence in comparison to others. In this study, they considered the following sports: aerobics, badminton, basketball, soccer, gymnastics, hockey, kick‐boxing, martial arts, rugby, running, skate‐boarding, and squash [2]. In addition to this, we also know that there are two main factors associated with this injury: the biomechanics of the foot referred to as foot pathologies or alterations such as pes cavus and sport maneuvers [38, 45]. On the other hand, an extrinsic variable factor is also associated, such as the maneuvers performed during sports practice such as jumping, acceleration, and changing direction.

It is worth mentioning then that although these factors are the most studied in the literature, it does not mean that they are decisive for whether or not the fracture of the fifth metatarsal due to stress appears. However, it does give us an idea of where the research should be directed or find possible gaps in it that can complement the information related to the injury described. In matters of foot biomechanics, some authors have already carried out research trying to find what pathology or morphology can influence the plantar pressure overload in the area of the fifth metatarsal. Although this is not a parameter that guarantees that a fracture will occur in the future, it is an adequate measurement to infer that it may be a risk factor [17].

As for the other factor (extrinsic variable), we can mention that the research carried out concludes that these sports maneuvers can be an important risk factor for producing the fifth metatarsal fracture [27]. Miyazaki et al. carried out an analysis of how the athlete, when executing these sports maneuvers, increases the distribution of plantar pressure in the area of the fifth metatarsal, and using the finite element method, transfers that pressure to forces in the bone, which may or may not reach to exceed the yield limit of the material causing a stress fracture [27].

In a deeper analysis of the research made by Miyazaki et al. [27] we can identify that although the kinematic parameters of the foot are measured to generate the model using the finite element method, there is still an area of opportunity to explore the kinematics of the whole‐body structure and how this can influence the plantar pressure distribution of the fifth metatarsal area. In other words, does the kinematics of the body structure during the execution of sports maneuvers such as jumping, acceleration, and change of direction influence the plantar pressure distribution of the fifth metatarsal area? In such a way, this could be a risk factor associated with the appearance of the fracture. Saita et al. [40] described that the internal rotation of the hip could be considered as a risk factor for the appearance of the fifth metatarsal fracture. We believe that the kinematics of the body structure is associated with a greater risk of developing a stress fracture of the fifth metatarsal, since it is a modifiable factor, by measuring, monitoring, and improving it, we can prevent the occurrence of this injury.

Likewise, highlights certain limitations of the down available current scientific evidence, consequence to the heterogeneous methods applied in these original studies that including in this systematic research, which did not offer the opportunity to carry out a meta‐analysis. In addition, in future investigations, it should also be examined how these biases might affect the reliability of their results and, consequently, the validity of the pooled findings in this review.

Finally, the present systematic review gives helpful information to fifth metatarsal fracture researchers and podiatrist clinicians that investigate regarding the implementations of effective treatments and prevention strategies.

## 5 CONCLUSIONS

This research therefore shows that in terms of the incidence of fifth metatarsal fracture, it is due to multiple risk factors at work when trying to find the epidemiology of this injury occurring in the athletic population. In conclusion, the scoping review on fractures of the fifth metatarsal has provided valuable information on the prevalence, treatment, and complications of these fractures in athletes and the general population. However, there are challenges in the management and treatment of these fractures, as well as areas of opportunity for research in identifying risk factors and preventing them. More research is needed to improve the understanding of these fractures and develop more effective treatment and prevention strategies.

## AUTHOR CONTRIBUTIONS

**Luis Angel Ortiz‐Lango**: Conceptualization; data curation; formal analysis; investigation; methodology; supervision; writing—original draft; writing—review & editing. **Israel Miguel‐Andrés**: Conceptualization; formal analysis; investigation; methodology; supervision; writing—original draft; writing—review & editing. **Daniel López‐López**: Conceptualization; formal analysis; investigation; methodology; supervision; writing—original draft; writing—review & editing. **José de Jesús Mayagoitiza‐Vázquez**: Conceptualization; formal analysis; investigation; methodology; supervision; writing—original draft; writing—review & editing. **Ricardo Becerro‐de‐Bengoa‐Vallejo**: Conceptualization; formal analysis; investigation; methodology; supervision; writing—original draft; writing—review & editing. **Marta Losa‐Iglesias**: Conceptualization; formal analysis; investigation; methodology; supervision; writing—original draft; writing—review & editing. **Juan Gómez‐Salgado**: Conceptualization; formal analysis; investigation; methodology; supervision; writing—original draft; writing—review & editing. **Miguel Ángel Saavedra‐García**: Conceptualization; formal analysis; investigation; methodology; supervision; writing—original draft; writing—review & editing.

## CONFLICT OF INTEREST STATEMENT

The authors declare that they have no competing interests.

## ETHICS STATEMENT

Ethical approval was not required for this analysis.

## Data Availability

Author elects to not share the data. Research data are not shared.
